# Identification of a Three-Gene Signature Based on Epithelial-Mesenchymal Transition of Lung Adenocarcinoma Through Construction and Validation of a Risk-Prediction Model

**DOI:** 10.3389/fonc.2021.726834

**Published:** 2021-10-21

**Authors:** Jianguang Shi, Zishan Wang, Jing Guo, Yingqi Chen, Changyong Tong, Jingjie Tong, Wentao Hu, Chenwei Li, Xinjian Li

**Affiliations:** Thoracic Surgery Department, Ningbo First Hospital, Ningbo, China

**Keywords:** epithelial-mesenchymal transition, lung adenocarcinoma, prognosis, LASSO, risk score

## Abstract

Epithelial-mesenchymal transition (EMT) process, which is regulated by genes of inducible factors and transcription factor family of signaling pathways, transforms epithelial cells into mesenchymal cells and is involved in tumor invasion and progression and increases tumor tolerance to clinical interventions. This study constructed a multigene marker for lung predicting the prognosis of lung adenocarcinoma (LUAD) patients by bioinformatic analysis based on EMT-related genes. Gene sets associated with EMT were downloaded from the EMT-gene database, and RNA-seq of LUAD and clinical information of patients were downloaded from the TCGA database. Differentially expressed genes were screened by difference analysis. Survival analysis was performed to identify genes associated with LUAD prognosis, and overlapping genes were taken for all the three. Prognosis-related genes were further determined by combining LASSO regression analysis for establishing a prediction signature, and the risk score equation for the prognostic model was established using multifactorial COX regression analysis to construct a survival prognostic model. The model accuracy was evaluated using subject working characteristic curves. According to the median value of risk score, samples were divided into a high-risk group and low-risk group to observe the correlation with the clinicopathological characteristics of patients. Combined with the results of one-way COX regression analysis, HGF, PTX3, and S100P were considered as independent predictors of LUAD prognosis. In lung cancer tissues, HGF and PTX3 expression was downregulated and S100P expression was upregulated. Kaplan-Meier, COX regression analysis showed that HGF, PTX3, and S100P were prognostic independent predictors of LUAD, and high expressions of all the three were all significantly associated with immune cell infiltration. The present study provided potential prognostic predictive biological markers for LUAD patients, and confirmed EMT as a key mechanism in LUAD progression.

## Introduction

Lung adenocarcinoma (LUAD) is the most common subtype of non-small-cell lung cancer (NSCLC), with an increasing incidence and poor patient prognosis (1–3). LUAD is highly heterogeneous and aggressive, which is often associated with genetic mutations (4, 5). In recent years, advances in chemotherapy, radiotherapy, and targeted therapies have reduced LUAD mortality (6–8), but its long-term survival is still dismal when compared with other cancers (9). To the best of our knowledge, the molecular pathogenesis of LUAD remains largely unknown. Therefore, there is an urgent need to investigate the mechanisms of LUAD development in-depth and to search potential key prognostic markers.

In terms of primary tumor, spread of cancer cells resulted from metastasis is a main cause of lung cancer-related death (10). During tumor metastasis, local invasion of primary tumor affects only surrounding tissues, especially stromal cells, and shed tumor cells enter the circulatory system through endocytosis and survive in a fluid circulatory system environment. Tumor cells in circulatory system will in turn penetrate the circulatory system and migrate into distant tissues, forming tiny metastatic clones, which will then form visible metastatic foci through proliferation (11, 12). In such a tumor metastasis process, epithelial-mesenchymal transition (EMT) is a highly critical mechanism, through which epithelial cells lose cell-cell junctions and polarity, resulting in loss of epithelial properties and acquisition of mesenchymal properties with invasive and migratory abilities (13). EMT process is often activated during the development of tumorigenesis and growth, invasion, migration, colonization, and therapeutic resistance (14–16). In recent years, many studies have reported EMT as a marker of poor prognosis in LUAD patients (17, 18). Some studies showed the prognostic significance and biological functions of some EMT-related genes in LUAD (19), but such studies are more at an early stage and lack in-depth analysis.

In this study, we first collected EMT-related genes. Candidate genes were obtained through co-analysis with differentially expressed genes and total survival-related genes in LUAD. Then a gene signature prognostic risk prediction model was constructed by LASSO and COX regression analysis and validated. LUAD patients were divided into two groups of high and low risk according to risk scores, and prognostic significance of these gene signatures in LUAD was assessed through analyzing the correlation between different risk groupings and clinicopathological characteristics of patients.

## Methodology

### Data Collection

The Tumor Genetic Atlas (TCGA) database and Genotype-Tissue Expression (GTEx) database were downloaded to obtain human cancer gene expression data and clinical data. The information of LUAD samples and other 32 cancer types in TCGA, including clinically relevant pathological parameters and RNA-seq of tumor tissue samples, was assessed according to data completeness of clinical sample and degree of matching with sequenced samples remove duplicate and censored samples as well as cases without clinical outcomes. EMT-related genes were obtained from the Epithelial-Mesenchymal Transition (EMT) gene database (http://www.dbemt.bioinfo-minzhao.org/) download. The independent validation cohorts GSE68465 and GSE68571 were downloaded from the Gene Expression Omnibus (https://www.ncbi.nlm.nih.gov/geo) using the GEO query R package. Tumor name abbreviations and corresponding meanings are given in **Supplementary Table 1**.

### Differentially Expressed Genes

Differentially expressed genes in LUAD were screened using the edgeR package in R software with |logFC|≧1.5 and ADJUST P value<0.05. Volcano plots of the differentially expressed genes were drawn using ggplot2.

### Prognostic Risk Modeling and Analysis

LASSO regression was applied to identify genes and develop a gene signature. The effect of gene signature on prognosis was validated by one-way COX regression. Multifactor COX regression was employed to detect prognostically independent predictors and construct a prognostic risk model. The risk score was calculated according to the model, and the median risk score was the threshold to divide patients into high-risk and low-risk groups. Kaplan-Meier survival curves were drawn to compare the survival of high-risk and low-risk patients. Also, the working curves of subjects were drawn, and the area under the curve was calculated to assess the predictive efficacy of the model.

### Survival Analysis

Univariate cox regression analysis was done, and forest plots through the “forestplot” R package were used to display the P value, HR, and 95% CI of each variable. Use R software v4.0.3 for statistical analysis. If not otherwise stated, the rank sum test detects two sets of data, and a P value of <0.05 is considered statistically significant.

### TIMER Analysis

The TIMER database (http://timer.comp-genomics.org/) was used to analyze the expression of genes in LUAD correlated with immune cell infiltration.

### Protein Expression Validation

Immunohistochemical staining maps of protein expression in both liver cancer tissues and normal tissues were downloaded from the HPA database (The Human Protein Atlas) for validating the gene signature.

### Gene-Set Enrichment Analysis

RNA-seq profiles were uploaded to GSEA to investigate key gene-related signaling pathways in the high-risk group and the low-risk group. The enriched sets were screened based on a FDR < 0.25 and P < 0.05 after 1,000 permutations.

### Cell Culture

The human bronchial epithelial cell line BEAS-2B and human LUAD cell lines NCI-H2009 were all purchased from Beyotime Biotechnology. All the cell lines were cultured in RPMI Medium 1640 (Gibco, USA) containing 10% fetal bovine serum (FBS) (Gibco, USA) and were incubated in a constant temperature incubator at 37°C and 5% CO_2_ for future use.

### Western Blot

The ice‐cold lysate buffer was added to extract cell lines proteins. After centrifugation, the supernatant was extracted (12,000 rpm, 4°C, 10 min), and the protein concentration was measured using a BCA Protein Assay Kit (Beyotime, China). A total of 20 µg protein was separated using 10% SDS/PAGE, and then transferred to the membrane by wet transfer. The membrane was blocked with 5% skimmed milk powder. The primary antibodies of HGF (Abcam, 1:200), PTX3 (Abcam, 1:1,000), S100P (Abcam, 1:1,000), GAPDH (Abcam, 1:1,000) were added into membrane and then incubated overnight in shaking bed at 4℃. Tris‐buffered saline with Tween 20 was used to wash the membrane for three times, 10 min each time. The secondary antibody (Goat Anti-Rabbit IgG, Abcam, 1:2,000) was subsequently added and incubated for 2 h at room temperature. Finally, protein bands were detected using the ECL Western Blot Detection Kit (Beyotime, China), and β‐actin was used as the internal reference protein.

## Results

### Differentially Expressed Gene Screening

**Figure 1:**
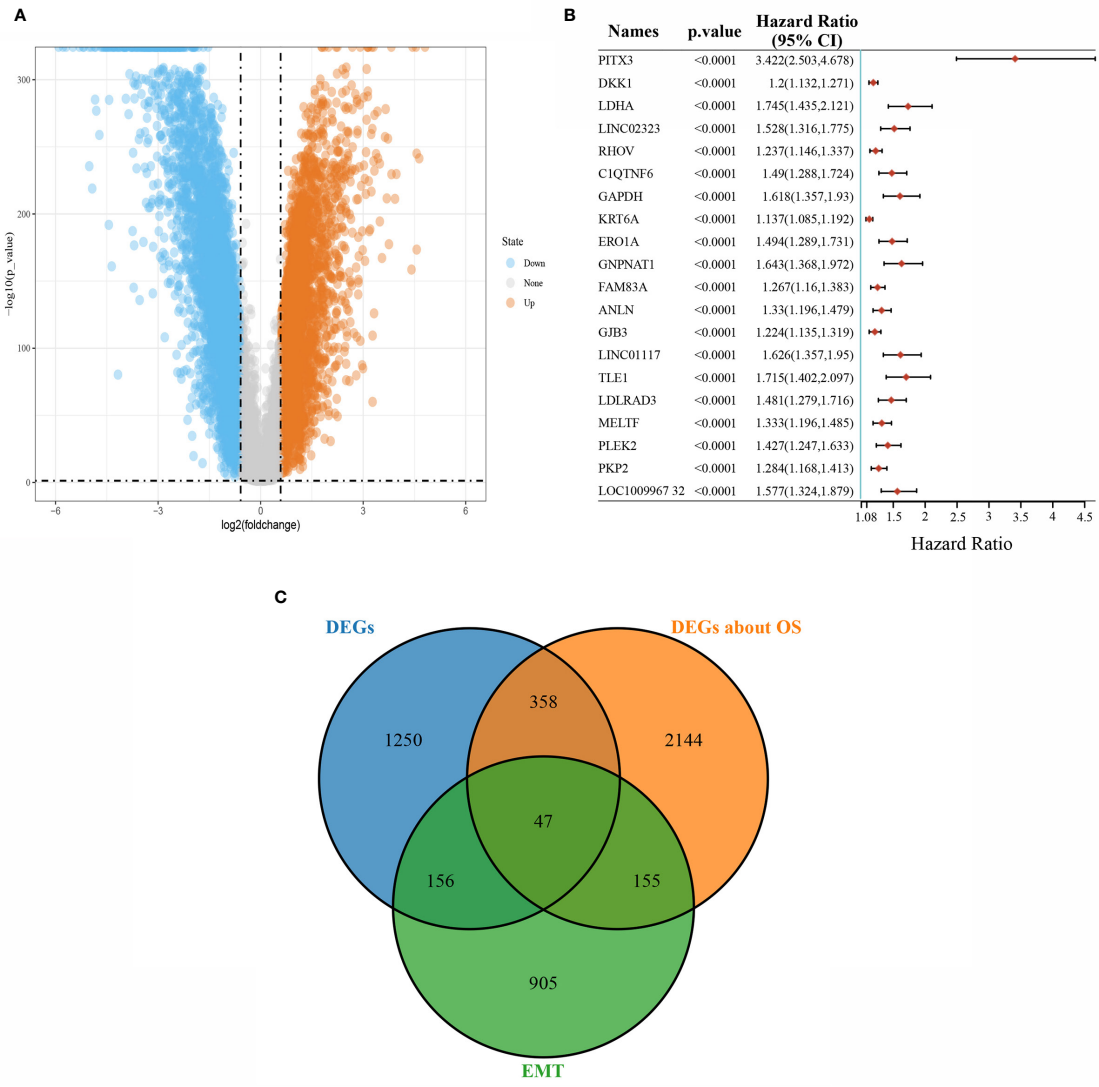
Screening of differentially expressed genes. **(A)** Volcano plot showing differentially expressed genes in LUAD; **(B)** The top 20 total survival-related genes in LUAD; **(C)** Venn diagram of differentially expressed, survival-related, and EMT-related overlapping genes.

Under the conditions of |logFC|>1 and P<0.05 as the screening conditions, a total of 1,811 differentially expressed genes, including 1,200 downregulated genes and 611 upregulated genes, were obtained from TCGA-LUAD data. The visualization results are shown in the volcano plot (**Figure 1A**). The prognostic characteristics of the top 20 genes with the most significant single-factor COX analysis in LUAD are shown in **Figure 1B**. The Epithelial-Mesenchymal transition (EMT) gene database (http://www.dbemt.bioinfo-minzhao.org/) was used to obtain 1,263 EMT-related genes, and the three categories of genes with overlaps were screened to obtain a total of 47 key genes (**Figure 1C**).

### Prognostic Prediction Modeling

**Figure 2:**
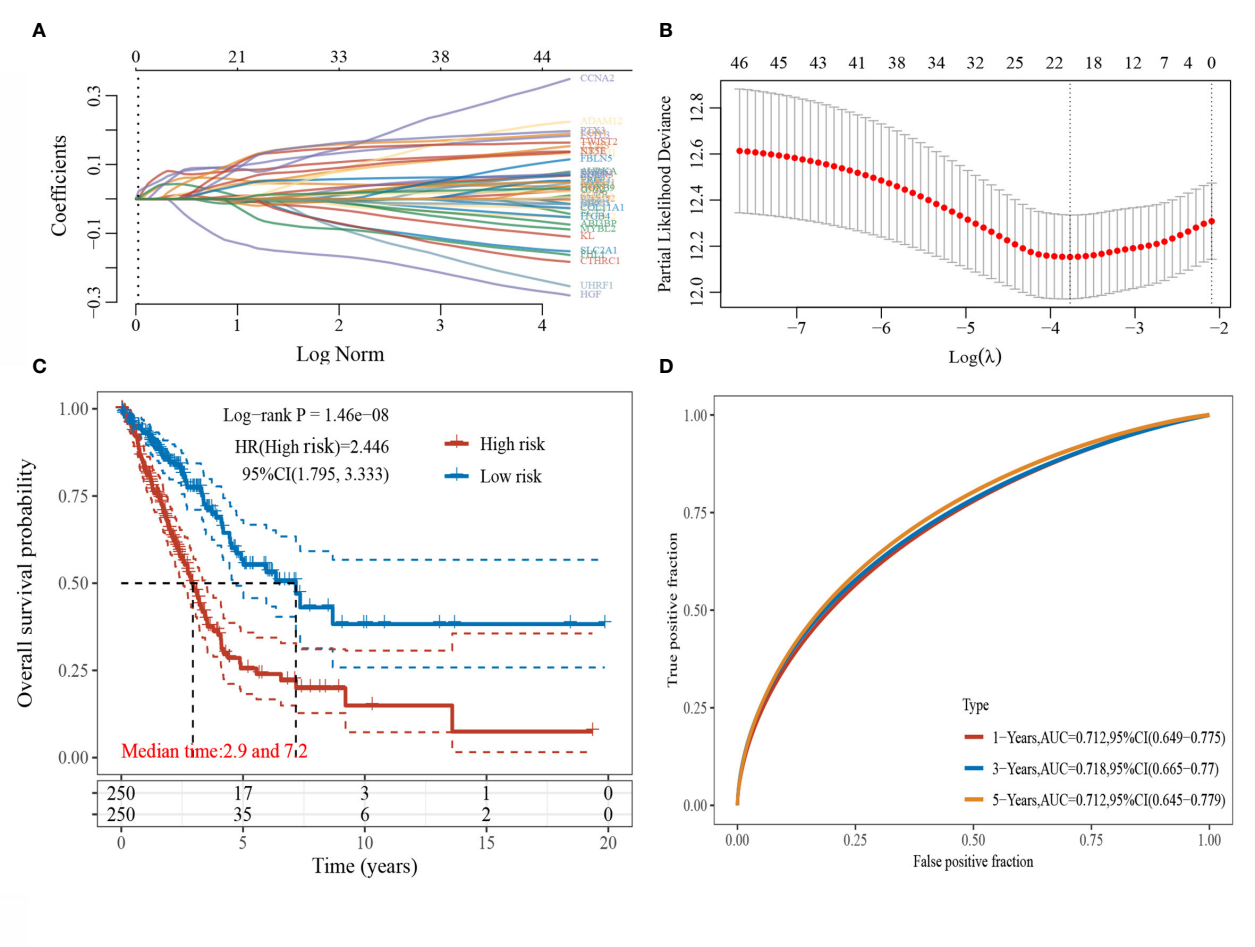
LASSO regression analysis. **(A)** Coefficients of selected characteristics are shown by lambda parameters; **(B)** Partial likelihood deviation plotted against log(l) using LASSO-Cox regression model; **(C)** Kaplan-Meier survival plots of high and low risk patients; **(D)** Time-dependent ROC analysis the gene signature.

LASSO regression analysis was conducted to further narrow the range of prognosis-related genes to ensure the result stability (**Figures 2A, B**). The risk score for each sample was calculated using the risk score formula:


$$\begin{array}{c} Riskscore=(0.0822)*KRT8+(0.0563)*ADM+(0.0099)*ECT2\\ +(0.0869)*CCNA2+(0.0418)*TYMS+(0.0951)*FSTL3\\ +(0.0374)*GOLM1+(0.0083)*EGLN3+(0.1054)*LGR4\\ +(-0.1263)*HGF+(-0.006)*KL+(0.0367)*S100P\\ +(-0.013)*AGER+(0.0513)*NT5E+(-0.0113)*NDRG2\\ +(-0.0022)*GPC3+(0.0034)*CA9+(0.0975)*TWIST2\\ +(-0.0198)*FHL1+(0.0829)*PTX3 \end{array}$$

The median risk score served as the threshold value to divide the patients into high-risk and low-risk groups. The survival curves were plotted (**Figure 2C**), and the results showed that the survival prognosis of high-risk patients was significantly worse than that of low-risk patients. The ROC curves of this risk score model in predicting the 1-, 3-, 5-year survival of LUAD patients were plotted (**Figure 2D**), and the AUC areas were 0.712, 0.718, and 0.712, respectively, indicating that this risk model had a high prediction accuracy.

### Clinicopathological Characteristics of Different Risk Score Subgroups

**Figure 3:**
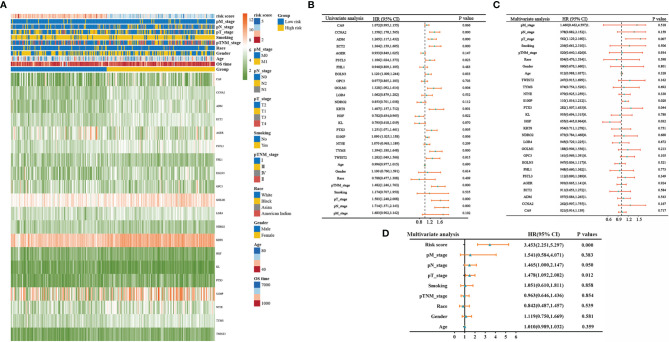
Clinicopathological characteristics of different risk score groupings. **(A)** Heatmap showing clinicopathological characteristics of different risk score subgroups; **(B)** forest plot showing single-factor COX regression analysis gene signature; **(C)** forest plot showing multifactor COX regression analysis gene signature; **(D)** forest plot showing multifactor COX regression analysis risk score.

Details of the distribution of clinicopathological characteristics of different risk score subgroups (high-risk and low-risk) are shown in a heatmap (**Figure 3A**), and the details are shown in **Table 1**. The results of univariate and multifactorial COX regression analyses displayed that risk score, TNM stage, T stage, S100P, HGF, and PTX3 could be used as independent predictors of prognosis of patients with LUAD (**Figures 3B**–**D**).

**Table 1:** Clinical characteristics of patients in high- and low-risk subgroups (TCGA, n=283).

Characteristics	Number (High/Low-risk group)	Proportion of patient
**Age (years)**		
≧65	74/76	53.0%
<65	76/57	47.0%
**Sex**		
Female	76/74	53.0%
Male	74/59	47.0%
**Race**		
White	136/114	88.3%
Black	11/16	9.5%
Asian	2/3	1.8%
American Indian	1/0	0.4%
**Smoking**		
Yes	126/111	83.7%
No	24/22	16.3%
**Stage**		
i	67/82	52.6%
II	42/27	24.4%
III	31/16	16.6%
IV	10/8	6.4%
**T stage**		
T1	42/50	32.5%
T2	89/68	55.5%
T3	15/8	8.1%
T4	4/7	3.9%
**N stage**		
N0	84/101	65.4%
N1	37/19	19.8%
N2	29/13	14.8%
**M stage**		
M0	140/125	93.6%
M1	10/8	6.4%

### Validating the Prediction of the Risk Model in Independent Cohort

**Figure 4:**
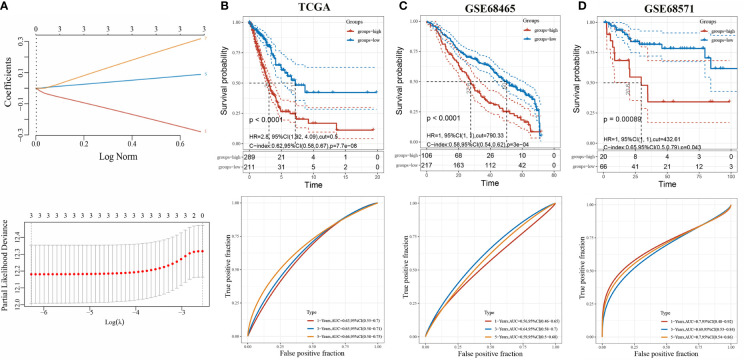
Evaluation of three-gene risk model performance in independent datasets. **(A)** LASSO-Cox regression model based on three genes; **(B)** Kaplan-Meier curves, univariate Cox regression of overall survival, and ROC curve analyses in TCGA; **(C)** Kaplan-Meier curves, univariate Cox regression of overall survival, and ROC curve analyses in GSE68465; **(D)** Kaplan-Meier curves, univariate Cox regression of overall survival, and ROC curve analyses in GSE68571.

We constructed a prognosis predicting risk model based on HGF, PTX3, and S100P base on TCGA datasets (**Figure 4A**). The risk score for each sample was calculated with the risk score formula:


Riskscore=(-0.2793)*HGF+(0.0885)*S100P+(0.3172)*PTX3

The optimal cutoff risk score served as the threshold to divide the patients into high-risk and low-risk groups. The survival curves were plotted, and the results showed that the survival prognosis of high-risk patients was significantly worse than low-risk patients (**Figure 4B**). To further assess the prediction of the risk model, GSE68465 and GSE68571 derived from GEO database were employed as validation cohorts. Cox regression analysis and Kaplan-Meier curve demonstrated that the prognosis of high-risk patients was worse than low-risk patients, which was consistent with the results found in the TCGA-LUAD cohort (**Figures 4C, D**).

### Expression of HGF, PTX3, and S100P in Pan-Cancer and LUAD

**Figure 5:**
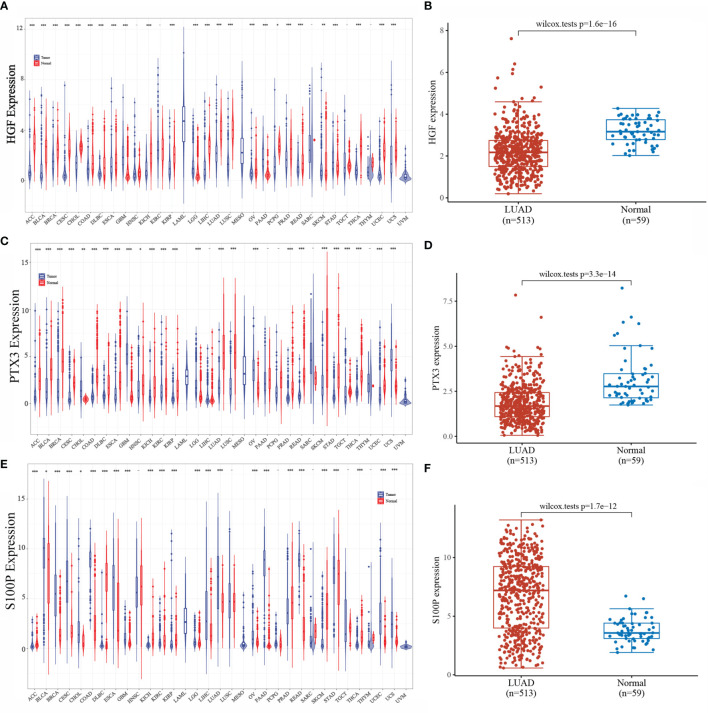
Expression of HGF, PTX3, and S100P. **(A)** Expression of HGF in pan-cancer; **(B)** Expression of HGF in LUAD; **(C)** Expression of PTX3 in pan-cancer; **(D)** Expression of PTX3 in LUAD; **(E)** Expression of S100P in pan-cancer; **(F)** Expression of S100P in LUAD; *P < 0.05, **P < 0.01, ***P < 0.001.

We analyzed the expression of HGF, PTX3, and S100P in tumors by integrating data from TCGA and GTEx database samples, and it was found that HGF and PTX3 expression was downregulated and S100P expression was upregulated in most tumors as compared with normal tissues (**Figures 5A, C, E**). TCGA database samples and integration of GTEx database normal samples displayed that in LUAD, HGF and PTX3 expressions were both downregulated and S100P expression was upregulated (**Figures 5B, D, F**).

### Survival Analysis of HGF, PTX3, and S100P

**Figure 6:**
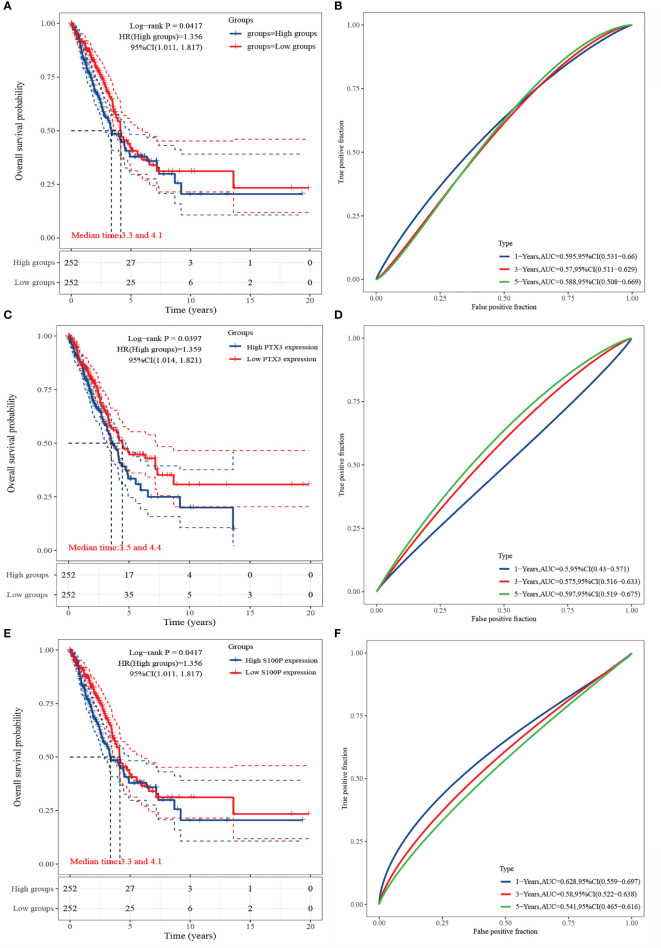
Survival analysis of HGF, PTX3, and S100P. **(A)** KM curve of HGF; **(B)** ROC curve of HGF; **(C)** KM curve of PTX3; **(D)** ROC curve of PTX3; **(E)** KM curve of S100P; **(F)** ROC curve of S100P.

The results of Kaplan-Meier analysis showed (**Figures 6A, C, E**) that high and low expressions of HGF, PTX3, and S100P were significantly correlated with patient prognosis. Specifically, high expression of HGF, PTX3, and S100P were all considered as poor prognostic factors in LUAD patients; however, ROC curves showed that all the three were less accurate when predicting the prognosis (**Figures 6B, D, F**).

**Figure 7:**
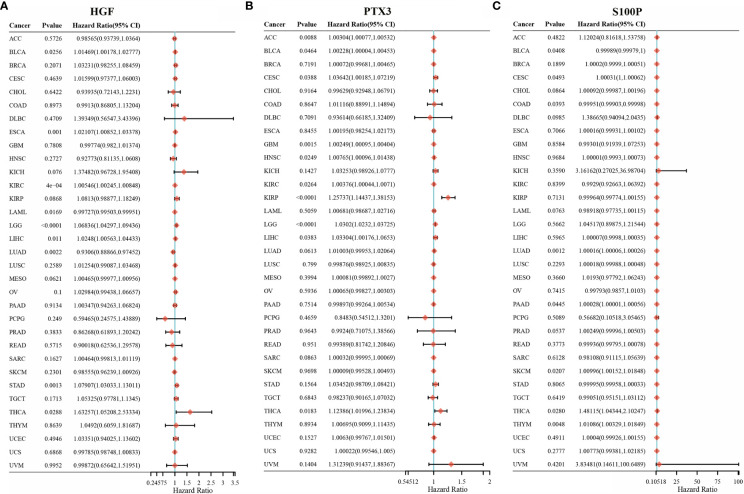
HGF, PTX3, S100P on the prognosis of other cancers. **(A)** Forest plot of HGF on the prognosis in 33 cancer types; **(B)** Forest plot of PTX3 on the prognosis in 33 cancer types; **(C)** Forest plot of S100P on the prognosis in 33 cancer types.

We further investigated the association of HGF, PTX3, and S100P expression with overall survival in 33 tumors *via* univariate survival analysis. As shown in **Figure 7**, HGF could significantly affect the overall survival of BLCA, ESCA, KIRC, LGG, LIHC, STAD, THCA (**Figure 7A**); PTX3 could noticeably affect the overall survival of ACC, BLCA, CESC, GBM, HNSC, KIRC, LGG, LIHC (**Figure 7B**); S100P could significantly affect the overall survival of CESC, LUAD, THCA, THYM (**Figure 7C**).

### Correlation of HGF, PTX3, and S100P With Immune Cell Infiltration

**Figure 8:**
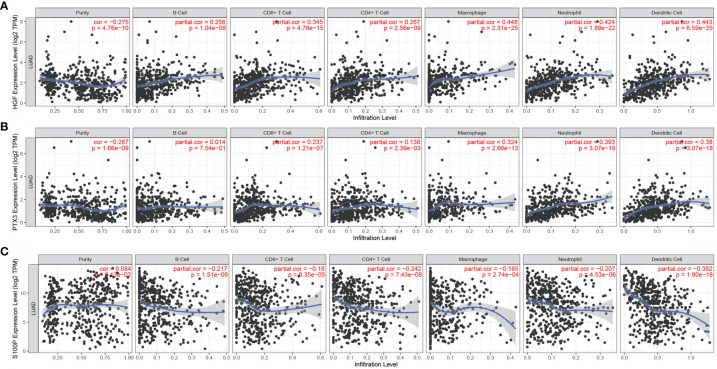
Correlation of HGF, PTX3, and S100P with immune cell infiltration. **(A)** Correlation between HGF and immune cell infiltration; **(B)** Correlation between PTX3 and immune cell infiltration; **(C)** Correlation between S100P and immune cell infiltration.

The expression levels of HGF in LUAD were significantly positively correlated with tumor purity, B cells, CD8+ T cells, CD4+ T cells, neutrophils, and dendritic cells (**Figure 8A**). However, the expression levels of PTX3 in LUAD were significantly negatively correlated with tumor purity and positively correlated with B cells, CD8+ T cells, CD4+ T cells, neutrophils, and dendritic cells (**Figure 8B**). The expression level of S100P was negatively correlated with B cells, CD8+ T cells, CD4+ T cells, neutrophils, and dendritic cells but positively correlated with tumor purity in LUAD (**Figure 8C**).

### Validation of HGF, PTX3, and S100P Protein Expression

**Figure 9:**
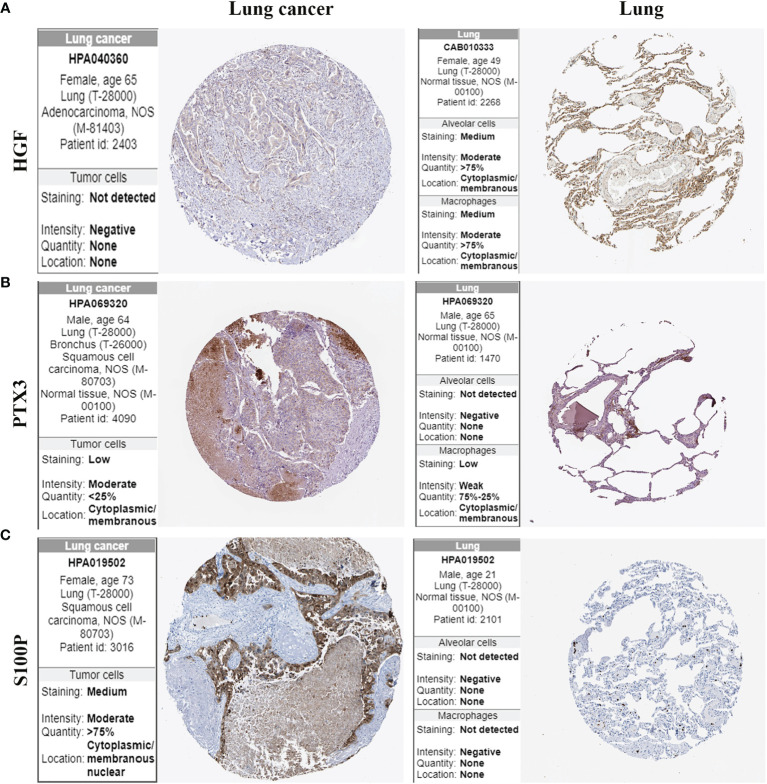
Protein expression of HGF, PTX3, and S100P in lung cancer tissues and normal lung tissues. **(A)** HGF protein expression; **(B)** PTX3 protein expression; **(C)** S100P protein expression.

The protein expression of HGF, PTX3, and S100P in lung cancer tissues and normal lung tissues was validated using the HPA online database. The results demonstrated that HGF was expressed in normal lung tissues but it was not detected in lung cancer tissues (**Figure 9A**). PTX3 was mildly expressed in lung cancer tissues but was not detected in normal alveolar cells (**Figure 9B**). S100P was moderately expressed in lung cancer tissues but was not detected in normal lung tissues (**Figure 9C**).

### Gene Set Enrichment Analysis

**Figure 10:**
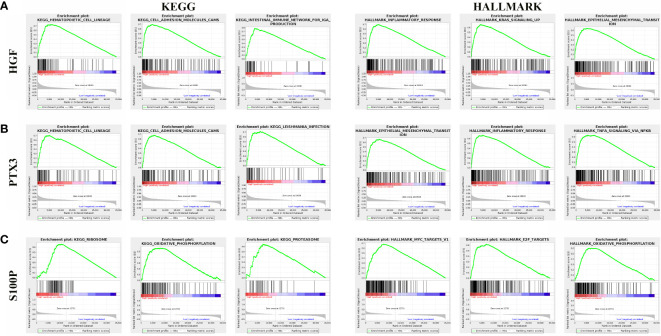
Gene set enrichment analysis of three genes associated with signaling pathways in KEGG and hallmark datasets. **(A)** Results of GSEA of HGF ranked in the top three for its correlation with signaling pathways in KEGG and HALLMARK database. **(B)** Results of GSEA of PTX3 ranked in the top three for its correlation with signaling pathways in KEGG and HALLMARK database. **(C)** Results of GSEA of S100P ranked in the top three for its correlation with signaling pathways in KEGG and HALLMARK database.

To examine the effect of gene expression on tumors, we divided the human pan-cancer samples into two groups with high and low expression, according to the expression of HGF, PTX3, S100P, and the enrichment of signaling pathways in KEGG and HALLMARK in high- and low-expression groups was analyzed by GSEA. The top three signaling pathways most significantly enriched in both databases have been listed. GSEA verified that HGF was mainly enriched in hematopoietic cell lineage and inflammatory response (**Figure 10A**), and that PTX3 was mainly enriched in hematopoietic cell lineage and epithelial mesenchymal transition (**Figure 10B**), and that S100P was mainly enriched in ribosome and MYC target (**Figure 10C**).

### *In Vitro* Experiments

**Figure 11:**
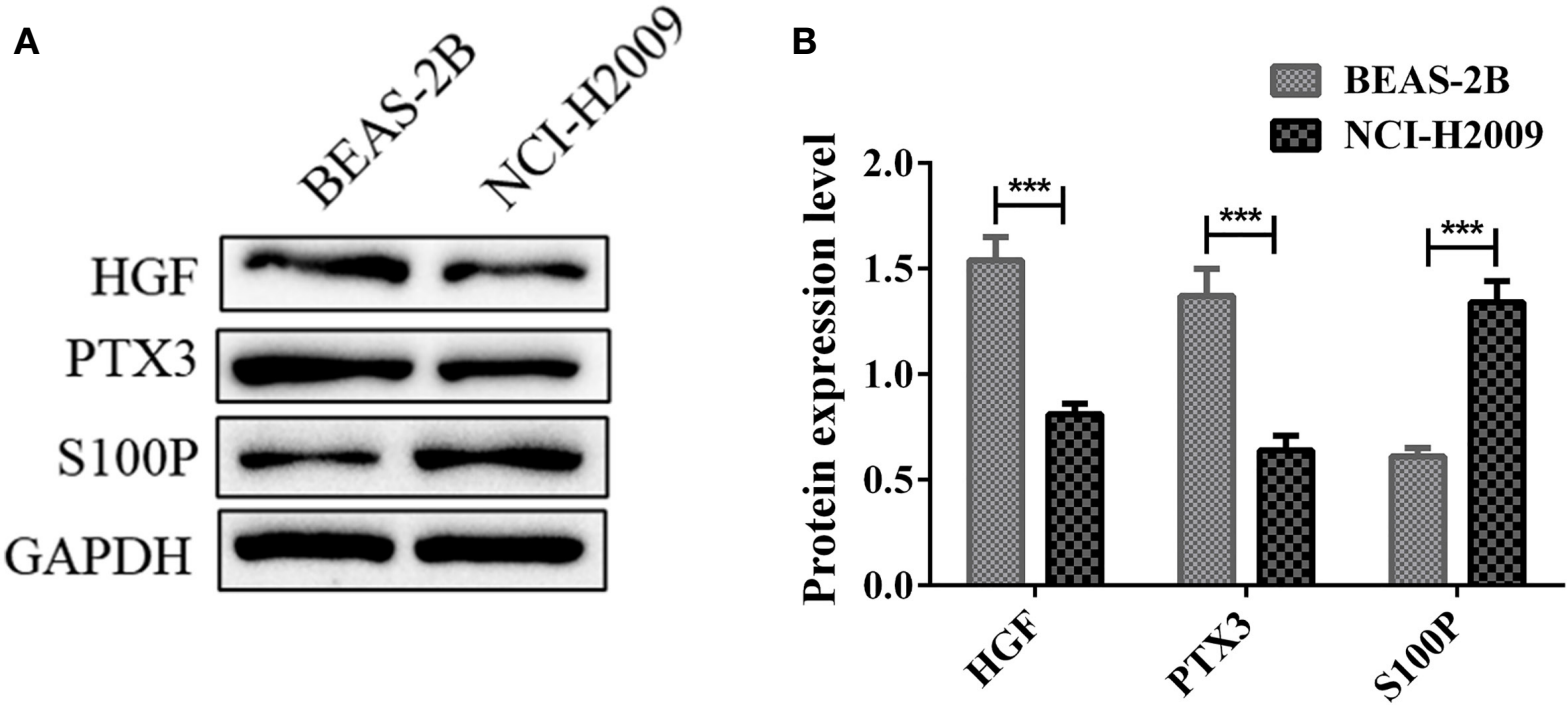
Protein expression levels of HGF, PTX3, S100P in BEAS-2B and NCI-H2009. **(A)** Western blot bands representing HGF, PTX3, S100P protein expression level in BEAS-2B and NCI-H2009; **(B)** Protein expression level of HGF, PTX3, S100P. ***P < 0.001.

We further verified the protein expression level of HGF, PTX3, S100P *in vitro*. Western blotting results showed that the protein expression level of HGF and PTX3 were downregulated in NCI-H2009 *versus* BEAS-2B, while S100P was upregulated (**Figure 11**).

## Discussion

Epithelial-mesenchymal transition (EMT) is a fundamental and critical cell biological process during embryonic development (20). EMT is a reversible process, with the reversal being referred to as mesenchymal-epithelial transition (MET). EMT and MET are essential for embryonic development, tissue regeneration, and wound healing, and its role in contributing to organ fibrosis, cancer progression, and metastasis requires a full understanding (21, 22). EMT confers tumor cells the ability to detach from the primary tumor mass and invade lymphatic vessels and blood vessels through extracellular matrix, allowing them to reach secondary tissues or distal organs and reactivate epithelial cell properties to form metastatic foci at secondary sites *via* the MET pathway (23). Assessing EMT state in tumors is challenging as the process is transient and reversible (24). Previous studies have focused on exploring the biological functions and molecular mechanisms of EMT-related genes, such as the expression of E-cadherin, claudins, ocludins, and cytokeratins, and considered these genes as common markers of epithelial state, and Vimentin (VIM), fibronectin, and α-SMA as the markers of mesenchymal state (25–27). However, for some circulating tumor cells (CTCs) underwent EMT, the expression of these (25–27) epithelial cell adhesion-based molecular markers is difficult to be detected (28). Immunicon, an FDA-approved company, has demonstrated that the number of CTCs is indicative of patient prognosis, and Tang et al. (19) first developed a new EMT-related gene signature and constructed a nomogram to predict prognosis of LUAD patients. In addition, the construction of predictive models based on EMT-related genes in LUAD has not been reported. Therefore, searching prognostic predictive biomarkers for LUAD patients based on EMT-related genes may be a promising approach.

In this study, we identified three prognosis-related gene signatures, namely, HGF, PTX3, and S100P. We first constructed a prognostic prediction risk model based on 20 EMT-related gene signatures and verified that the model had strong prediction accuracy through KM curve and ROC curve. Patients were grouped according to their risk score differences. HGF, PTX3, S100P, and risk score were all identified as independent predictors of prognosis of LUAD patients through performing univariate and multifactorial COX regression analyses on these 20 genes and risk scores. The results showed that hGF and PTX3 expression was downregulated and S100P expression was upregulated in LUAD, and that the expression of all the three was correlated with immune cell infiltration, suggesting that all the three gene could promote tumor progression. HGF, which is a cytokine produced by mesenchymal fibroblasts (29), could stimulate the migration, proliferation, migration, cell survival, morphogenesis, and angiogenesis of epithelial cells (30). It has been found that the HGF/c-Met signaling pathway may influence multiple aspects of tumor development by activating specific pathways that induce interactions between cancer cells and the tumor microenvironment in which they reside (29). For example, in EGFR-mutant lung cancer, HGF may be involved in endogenous and acquired resistance to acid kinase inhibitors (31). GSEA results also demonstrated that HGF was mainly enriched in inflammatory response. PTX3 in the pentraxin family is an immunomodulatory factor involved in angiogenesis, proliferation, and immune escape in cancer (32). It has been reported that PTX3 tends to be expressed in the stroma rather than in tumor cell components, suggesting that the PTX3 gene is epigenetically modified and silenced in cancer cells (33). This is similar to our findings, as we found that PTX3 was low-expressed in tumor cells by protein expression. S100P is a member of the S100 calcium-binding protein. S100P in the S100 calcium-binding protein family is originally isolated from human placenta (34), and S100P expression is elevated in a variety of tumor cell lines and tumor tissues, including in lung cancer (35), pancreatic cancer (36), and breast cancer (37). Moreover, it is also a tumor microenvironment-associated gene (38). Our western blotting results also demonstrated that the protein expression level of HGF and PTX3 were upregulated in NCI-H2009 cell line compared to BEAS-2B cell line, while the protein expression level of S100P was downregulated.

There are some limitations to this study. Firstly, LUAD is a highly heterogeneous tumor, but certain key clinical variables were not available in public databases, which limited the comprehensiveness when developing the prognostic model. Furthermore, the predictive ability of the EMT-related gene signature using these three genes was only based on bioinformatics analysis, and more basic experiments and clinical evidence are needed to validate the signature.

In summary, this study constructed a prognostic model based on 20 EMT-related gene signatures and validated the performance the model. The final three prognostic predictors of LUAD patients were analyzed by COX regression analysis to obtain three independent factors. These findings may provide a new direction for prognosis prediction and individualized treatment for patients with LUAD.

## Data Availability Statement

The original contributions presented in the study are included in the article/**Supplementary Material**. Further inquiries can be directed to the corresponding authors.

## Author Contributions

All authors listed have made a substantial, direct, and intellectual contribution to the work and approved it for publication.

## Conflict of Interest

The authors declare that the research was conducted in the absence of any commercial or financial relationships that could be construed as a potential conflict of interest.

## Publisher’s Note

All claims expressed in this article are solely those of the authors and do not necessarily represent those of their affiliated organizations, or those of the publisher, the editors and the reviewers. Any product that may be evaluated in this article, or claim that may be made by its manufacturer, is not guaranteed or endorsed by the publisher.

## Supplementary Material

Supplementary Table 1 Tumor name abbreviations and corresponding meanings.
